# A Light-Weight Deep-Learning Model with Multi-Scale Features for Steel Surface Defect Classification

**DOI:** 10.3390/ma13204629

**Published:** 2020-10-16

**Authors:** Yang Liu, Yachao Yuan, Cristhian Balta, Jing Liu

**Affiliations:** 1 Bremen Institute for Mechanical Engineering-bime, University of Bremen, 28359 Bremen, Germany; sea.yang.liu@gmail.com; 2 Institute of Computer Science, University of Goettingen, 37077 Goettingen, Germany; 3 Faculty of Mathematics, University of Goettingen, 37077 Goettingen, Germany; c.baltarivera@stud.uni-goettingen.de; 4 Key Laboratory of Materials Processing Engineering, School of Material Science and Engineering, Xi’an Shiyou University, Xi’an 710065, China

**Keywords:** surface defect classification, multiple image scales, convolutional neural networks, classification accuracy, latency

## Abstract

Automatic inspection of surface defects is crucial in industries for real-time applications. Nowadays, computer vision-based approaches have been successfully employed. However, most of the existing works need a large number of training samples to achieve satisfactory classification results, while collecting massive training datasets is labor-intensive and financially costly. Moreover, most of them obtain high accuracy at the expense of high latency, and are thus not suitable for real-time applications. In this work, a novel Concurrent Convolutional Neural Network (ConCNN) with different image scales is proposed, which is light-weighted and easy to deploy for real-time defect classification applications. To evaluate the performance of ConCNN, the NEU-CLS dataset is used in our experiments. Simulation results demonstrate that ConCNN performs better than other state-of-the-art approaches considering accuracy and latency for steel surface defect classification. Specifically, ConCNN achieves as high as 98.89% classification accuracy with only around 5.58 ms latency over low training cost.

## 1. Introduction

Steel strips are critical products in iron and steel industries, the quality of the steel strips directly influences the appearance and quality of the final product [1]. To produce high-grade steel strips, massive efforts are taken to inspect surface defects emerging in the production process. There are different types of defects, e.g., scratch, patch, pitted surface, etc. The defects on steel surfaces can cause bad appearance, weaker strength, corrosion, and increased friction in practice, consequently resulting in economic loss in forging industries. Therefore, it is paramount for the iron and steel industries to be able to inspect and detect defects accurately in real time.

Since the 1990s, defect detection and classification has been studied. The methods are mainly flux leakage testing and artificial visual inspection, which are time-consuming, labor-intensive, and expensive. With the development of computing and programming tools, traditional image processing-based and machine learning methods have been employed to detect surface defects. Traditional image processing-based methods include threshold-based methods, wavelet transform models, and Gaussian mixture entropy models [2] which use primitive attributes to discover defects. However, these methods are not robust on noisy real-world images/videos, and cannot meet real-time demand. Additionally, manually selected features are extracted in these methods which subjectively affect the classification reliability. The performance of machine learning methods heavily relies on the feature generation process, while obtaining high-quality features is difficult and requires expertise.

With the recent advancement in deep learning, surface defect classification with deep learning-based techniques has become popular. For example, the works of [3,4,5] utilize deep learning models for surface defects classification which proves that deep learning models are far more accurate than traditional image processing-based and machine learning methods. To enhance the performance, data augmentation methods are usually utilized [6]. The generated images with or without defects further improves detection accuracy. To improve the accuracy of defects classification when there is few labeled data and a lot of unlabeled data, semi-supervised learning method like [7] is developed to take advantage of the unlabeled data, which are easier to obtain than labeled data, with good performance. Moreover, feature fusion and context attention strategies are also employed to achieve better accuracy [3], which particularly address the problem of low performance on images with large intra-class inconsistency and inter-class similarity.

Despite the outstanding advantages of deep learning models, there is still a fatal drawback, i.e., large precisely labeled datasets and high-performance computers are needed to achieve acceptable classification accuracy, high training cost due to a large number of unlabeled data for training process in semi-supervised method. Therefore, in this paper, a new method named Concurrent Convolutional Neural Network (ConCNN) is developed, which can automatically detect steel surface defects rapidly and accurately while only requires a small training dataset. ConCNN is a novel multi-scale CNN (Convolutional Neural Network) model leveraging the relationship of images with different scales for real-time surface defects classification. Specifically, features from images with different scales are extracted by a simple CNN first. The features are then integrated and classified. Various defect sizes are explored to discover the optimal combination. ConCNN can be utilized in both online and offline stages. In the online stage, ConCNN can predict and learn the incoming new defect types automatically. The performance of the proposed model is compared with the state-of-the-art models on a public dataset in terms of accuracy and latency.

The paper is organized as the following structure: Section 2 gives a comparison of some related representative methods for surface defect detection, in Section 3 some basic knowledge of CNN is introduced. Section 4 describes details of the proposed algorithm. In Section 5 the dataset and the evaluation of the proposed model are introduced. The results and discussion are finished in Section 6 followed by the conclusions in Section 7.

## 2. Related Work

The related works can be categorized into two categories, i.e., traditional surface classification approaches and emerging machine learning-based surface classification approaches, which are summarized and compared in this section.

### 2.1. Traditional Surface Classification Approaches

Traditional surface classification methods can be further classified into three classes: statistical-based, spectral-based, and model-based.

Statistical-based: This type of methods is generally based on the evaluation of the regular and periodic distributions of pixel intensities. Neogi et al. [8] considered a global dynamic percentile thresholding method over gradient images for various kinds of defects on steel strips. The adaptive threshold changes according to the number of the pixels on the specific region of gradient images. Ma et al. [9] developed a method based on multi-directional gray level fluctuation which is used to deal with surface photos with uneven illumination and achieved satisfying detection results for metal, wood, and wall surfaces. However, the detection accuracy could be low due to the existence of various texture. Fan et al. [10] proposed an algorithm based on deep learning to detect cracks on roads. In this paper, an adaptive thresholding method was employed to extract cracks, a good accuracy up to 99.92% was achieved. Cañero-Nieto et al. [11] proposed a model using multi-linear regression and neural networks based on thresholding theory. The camera used to take the photos were normal but special lighting was needed.

Spectral-based: To avoid noise and intensity variations in pixels, methods are developed based on spectral transform. Jeon et al. [12] developed a dual-light switching-lighting method with sub-optimal filtering technology which makes the detection of defects on steel surfaces easier by turning defects into alternated black and white patterns. The method shows high accuracy but highly dependent on hardware. Thus, it is not suitable for large size images and low-performance computers. To reduce noise in rails images taken by unmanned aerial vehicles, Wu et al. [13] proposed a method combining wavelet transformation with median filtering. Their experiments prove that the influence of noise can be eliminated and achieved good performance. To detect defects in non-periodical patterns like printed circuit boards, Tsai et al. [14] developed a global Fourier image reconstruction method, which is proved robust to translation and changing of illuminations, and able to detect tiny defects up to 1 pixel wide.

Model-based: To overcome the limitations of the above-mentioned methods, model-based methods were explored. They can achieve better performance by projecting the initial texture distribution of the photos to a low-dimensional distribution. Liu et al. [15] developed a new model based on Haar-Weibull-variance. In the model, local gradient magnitude is replaced by the Haar feature. Good performance can be found even with low contrast of images. Bumrungkun et al. [16] proposed a method for edge detection based on snake active contour models, which enables the extraction of fabric feature to detect defects on fabrics and achieves around 98.77% accuracy. Yang et al. [17] proposed an improved algorithm based on an active contour model together with a segmentation algorithm based on an edge-less active contour model. Defects with fuzzy boundaries and complex background can be detected accurately.

Despite the high performance of these technologies, the applications of them are constrained on images with homogeneous texture, or heavily dependent on expertise or high-performance hardware.

### 2.2. Emerging Machine Learning-based Surface Classification Approaches

Bcha et al. [18] proposed a vision-based algorithm in which cracks on concrete are detected using a Convolutional Neural Network (CNN)-based model. The model is highly resistant in some real-world situations, for example, with changing illuminations or shadows. Another work from them [19] developed a model to detect structural damage. The model is a region-based CNN with flexible size sliding windows. Despite the varying size or scale of images, the algorithm is still able to detect the defects on concrete surfaces with high efficiency. He et al. [5] developed a semi-supervised learning model combining a convolutional autoencoder and a semi-supervised generative adversarial network to detect steel surface defects. The model obtained a 16% improvement compared with the traditional method for hot-rolled plates detection. Wang et al. [4] proposed a deep learning model for surface defects classification. The core of the model is a simple feed-forward neural network including convolutional layers and pooling layers. Dong et al. [3] utilized the feature fusion and context attention strategies to address the complexity of surface defects in both intra-class and inter-class. Specifically, multi-scale features were fused by skip connections first. The adjacent resolution is further processed by the global context attention technique. A new defect classification system based on deep learning was proposed by [20], where multiple features of deep learning are fused into one feature. Then, the regions of interest are produced by a region-based network. Li et al. [21] used the Kirsch operator first to obtain anomalous regions. Based on that, a deep CNN and support vector machine technique were employed to extract the defect features and classify the defect types, respectively. Song et al. [22] proposed a method using a CNN to detect weak scratches and achieved high-performance with noisy images. Zheng et al. [7] developed a generic semi-supervised deep learning model for automated surface inspection which can achieve high performance with a small labeled training dataset. However, a large unlabeled dataset is needed in the training process.

In summary, most of the previous works such as [5,22], require large, high-quality, and precisely labeled datasets for training to gain high-performance, which, however, is difficult, labor-intensive, and costly to collect in industries.

## 3. Preliminaries

Deep learning models, mostly in the form of Convolutional Neural Networks (CNNs), have been widely used for image classification [4], object detection [20], and text understanding [23]. CNNs are well known due to the advantages like high accuracy, high efficiency (with few layers), and ease-to-use (end-to-end). The features of input images are extracted and learned by CNNs automatically. A typical CNN usually includes several layers, which process and abstract the input images. This helps CNN learn valuable information from a mass of data. The trained CNN can further be employed to detect defects and discover images/samples with unknown defect types. Each CNN can be expressed by two mathematical constructs. The first one is the Cells. By utilizing input, threshold, as well as initial state independently, the collected information from input images is encrypted into Cells. The other one is used to describe the neighboring Cells within a range of influence $S_{ij}\left(r\right)$ of radius *r*.

Given scalars including state $x_{ij}\left(t\right)$, threshold $z_{ij}\left(t\right)$, input $u_{ij}\left(t\right)$, and output $y_{ij}\left(t\right)$ of each isolated Cell $C_{ij}$, the state equation of a standard CNN can be represented by [24]:

$$\begin{array}{c} {\dot{x}}_{ij}\left(t\right)=\frac{\partial x_{ij}\left(t\right)}{\partial t}=-x_{ij}\left(t\right)+\sum_{k,l\in S_{ij}\left(r\right)}a_{kl}y_{kl}\left(t\right)+\sum_{k,l\in S_{ij}\left(r\right)}b_{kl}u_{kl}\left(t\right)+z_{ij}\left(t\right),\\ y_{ij}\left(t\right)=f\left(x_{ij}\left(t\right)\right)≜\frac{1}{2}\left(\right|x_{ij}\left(t\right)+1|-|x_{ij}\left(t\right)-1\left|\right)=\left\{\begin{array}{cc} 1, & x_{ij}\geq 1\\ x_{ij}, & |x_{ij}|<1\\ -1, & x_{ij}\leq -1 \end{array}\right. \end{array}$$

where i=1,2,...,M,j=1,2,...,N. *M* and *N* are the dimensions of the row and column of input image. For color images, $x_{ij}\in R^{3}$, to capture three fundamental colors, e.g., red, green, and blue.

The CNN requires an input, i.e., an image, whose dimensions are defined by width, height, and the number of channels. The input image is processed by a convolutional layer that computes the convolutional product between the input and a kernel. The result is further processed by a rectified linear unit (ReLU), which is an activation function that captures the non-linearity of the pixels, e.g., g(x)=max(0,x). After that, the result is taken by a Pooling layer which is usually used to reduce dimensions. Max pooling (i.e., take the maximum over a subset of input) is chosen here out of several pooling methods, for example, average pooling, max pooling, etc., due to its advantages of identifying sharp or bright features. Moreover, the resultant matrix of the pooling layer is turned into a vector by the fully connected layer that applies a linear transformation. Finally, the output of the last layer is used as input of the classification layer where a classification function such as logistic regression model, is utilized to output a vector whose dimension is the number of classes.

## 4. Defect Classification System

In this section, the design goals, the architecture of the proposed ConCNN model, and the mathematical rationale behind the design are illustrated.

### 4.1. Design Goals

The main design goals of ConCNN are listed as follows:

- Real time: Real-time defects classification is important for defects classification in industrial manufacturing. However, it is not easy to achieve in real-world environments due to many factors, like high-speed production lines, diversity defects types, and various sizes of detects.
- Automatic Learning: The characteristics of defects on steel strip (e.g., defect type and size) evolve over time. A classification model should be able to learn new incoming defect patterns automatically in real-time.
- Labelled data: Due to high labor and financial cost, labeling large datasets is difficult. A classification model should be able to learn defects’ characteristics well with only a limited number of labeled samples.
- Latency: Real-time defects classification is a time-intensive task, the latency and storage requirements are more strict than delay-insensitive tasks.
- Accuracy: Defect classification models should correctly predict multiple types of defects, i.e., with high accuracy.
- Storage cost: The captured steel strip images increase with the rising of produced steel strips every day resulting in the drastic increase of storage cost. The cost can be lessened by an efficient storage strategy. A classification model should achieve a good trade-off between resource utilization and accuracy.

### 4.2. ConCNN Architecture

ConCNN is designed to leverage the benefits of fusing different scales of defects. Large scale images can improve the classification performance of small defects on steel strips such as rolled-in scale and crazing; while small scale images can improve the performance of large defects, for example, scratches. ConCNN can be used in both online and offline phase. In the offline phase, the trained ConCNN can be utilized to predict the defect types. ConCNN in the online phase can be employed to detect the new incoming defects and continuously train themselves with the new predicted samples.

**Figure 1:**
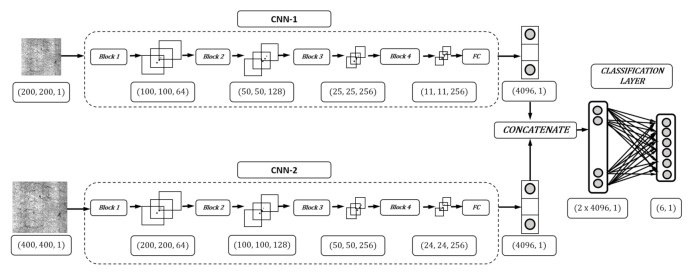
ConCNN architecture.

In Figure 1, ConCNN uses images with different scales as inputs. Two scales, i.e., (200, 200, 1) and (400, 400, 1), are unitized in ConCNN after extensively testing. Each input is processed by a sequence of blocks. CNN-1 process images with the dimension of (200, 200, 1) and CNN-2 process images with the dimension of (400, 400, 1). Both of CNN-1 and CNN-2 have the same structure and components but with different dimensions of input images. Besides, CNN-1 and CNN-2 works in parallel. Each CNN is a sequence of 4 blocks and a Fully Connected (FC) layer. There are mainly two layers in each Block, the “Convolutional layer” and “Pooling layer” as explained in Section 4.3. For example, the input images of CNN-1 are with the dimension of (200, 200, 1) and the output is a matrix with the dimension of (100, 100, 64). This output is the input of the next blocks and the final output is a matrix with the dimension of (11, 11, 256). The same rule applies to CNN-2. We have two different outputs from CNN-1 and CNN-2, i.e., a matrix with the dimension of (11, 11, 256) and a matrix with the dimension of (24, 24, 256), respectively. These matrices go through the FC layer which flattens the matrices to finally have two different vectors with the same dimension (4096, 1). Finally, these two vectors are concatenated and output a vector which is the input of the classification layer. The final classification output is a vector with the dimension of (6, 1) where each item is the log-likelihood of belonging each class.

**Figure 2:**
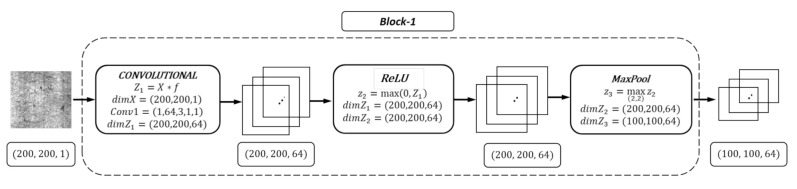
The detailed structure of a block.

In Figure 2, we present the detailed structure of block 1 as an example since all blocks in the proposed ConCNN model (as shown in Figure 1) have a similar structure, i.e., each block has a convolutional and Pooling layer. In this case, we start with the input which is an image with 200 × 200 pixels and one channel. After applying the convolutional layer, we have a matrix with the dimension of (200, 200, 64). After applying ReLU function, we obtain a matrix with the same dimension (200, 200, 64) but with only positive numbers. Finally, we apply the pooling layer to the last matrix with the dimension of (200, 200, 64) to reduce its dimensional to a matrix with the dimension of (100, 100, 64) which is the input of the next block.

### 4.3. Mathematical ConCNN

We present the mathematical explanation of the proposed ConCNN model for a better understanding. ConCNN consists of several steps described as follows:

- Input Image: The two types of images with different scales are utilized as inputs which can be denoted as:
  $$\begin{array}{c} I_{i}\left(h_{i},w_{i},c\right),i\in 1,2, \end{array}$$
  where $h_{i}$ is the number of rows of image scale/type *i*; $w_{i}$ is the number of columns of image type *i*; *c* denotes the number of channels.
- Convolutional layer: The 2D convolution process is applied to extract the features of inputs. In this process, larger images are reduced to matrices with smaller dimensions by calculating the convolutional product. We represent this layer as $Conv(I_{i}\left(h_{i},w_{i},c\right),C_{out},f,p,s)$. The computation of this layer gives a matrix $Z_{i}$ represented as $Z_{i}\left(m_{i},n_{i},C_{out}\right)$, which is defined as follows:
  (1) $$\begin{array}{c} Z_{i}=I_{i}*K. \end{array}$$
  The convolutional product * (i.e., the elements of matrix *Z*) is computed as:
  (2) $$\begin{array}{c} Z_{i}\left[m_{i},n_{i}\right]=\sum_{q=1}^{m_{i}}\sum_{r=1}^{n_{i}}I\left[q,r\right]\;\cdot \;K\left[m_{i}-q,n_{i}-r\right], \end{array}$$
  such that:
  (3) $$\begin{array}{c} m_{i}=\frac{h_{i}+2p-f}{s}+1, \end{array}$$
  (4) $$\begin{array}{c} n_{i}=\frac{w_{i}+2p-f}{s}+1. \end{array}$$
  where *q* and *r* are indexes of the sum of the convolutions problem.
  $I_{i}$ denotes the input Image of type *i*; *K* is the Kernel (also called filter) square matrix; *f* is the size of kernel matrix; $h_{i}$ is the number of rows of the input image of type *i*; $w_{i}$ is the number of columns of the output image of type *i*; *s* is the stride and it is the number of steps that the matrix K (Kernels) moves in each convolutions product process; *p* is the number of zeroes added to each side of the boundaries of the input, which is called padding; $m_{i}$ denotes the number of rows of the output image of type *i*; $n_{i}$ is the number of columns of the output image of type *i*; $Z_{i}$ is the output image of type *i*.
- ReLU: It is a layer identified by the activation function *g* that is used to introduce non-linearity to the network. ReLU stands for the Rectified Linear unit layer. We represent it as $ReLU(Z_{i}\left(m_{i},n_{i},C_{in}\right))$ and its computation gives another matrix g(Z) with same parameters. As it is defined in the next equation:
  (5) $$\begin{array}{c} g\left(Z_{i}\right)=max\left(0,Z_{i}\right), \end{array}$$
  where $Z_{i}$ denotes the input matrix of image type *i*; $g(Z_{i})$ is the output matrix of type *i*.
- Pooling layer: In this process, we reduce the size of the input image by taking features over a reduced subset of the input matrix, called pooling kernel. This layer also includes kernel, padding, and strides components. We represent this layer in a similar way as Conv layer, $Pool(g\left(Z_{i}\left(m,n,C_{in}\right),C_{out},f,p,s\right))$, and this layer gives a matrix $O_{i}\left(r_{i},c_{i},C_{out}\right)$, which is calculated as follows:
  (6) $$\begin{array}{c} O_{i}=\max_{\left(f,f\right)}g\left(Z_{i}\right). \end{array}$$
  such that,
  (7) $$\begin{array}{c} r_{i}=\frac{m_{i}+2p-f}{s}+1, \end{array}$$
  (8) $$\begin{array}{c} c_{i}=\frac{n_{i}+2p-f}{s}+1. \end{array}$$
  where $g(Z_{i})$ is the input image of type *i*; *f* is the size of kernel matrix; $m_{i}$ is the number of input image’s rows of type *i*; $n_{i}$ is the number of input image’s columns of type *i*; $r_{i}$ is the number of output image’s rows of type *i*; $c_{i}$ is the number of output image’s columns of type *i*; $O_{i}$ is the output matrix of type *i*.
- Fully-connected layer: This is a traditional perceptron neural network. This layer uses the previous pooling layer’s output as input.
  This fully connected layer is represented as $FC(O_{i}\left(r,c,C_{out}\right),l)$. First the matrix $O_{i}\left(r,c,C_{out}\right)$ is converted into a vector by the Vec(·) operator, which is give as Equation (9).
  (9) $$\begin{array}{c} Vec\left(O_{i}\right)=X_{i}. \end{array}$$
  Now, we have a vector *X* of dimension $\left(r\;\cdot \;c\;\cdot \;C_{out},1\right)$,
  (10) $$\begin{array}{c} Y_{i}=W_{i}X_{i}+b_{i}, \end{array}$$
  where $O_{i}$ is the input matrix of dimension (r,c); $W_{i}$ denotes the random matrix of dimension $\left(l,r\;\cdot \;c\;\cdot \;C_{out}\right)$; $b_{i}$ is the random vector of dimension (l,1); $Y_{i}$ is the output vector with dimension (l,1).
- Concatenation layer: Since we processed two types of images, the output of the previous layer gives us two types of vector $Y_{1}$ and $Y_{2}$ of a different dimension. We concatenate these two vectors in one vector *E* under the concatenate procedure. We represent it as $Concat(Y_{1},Y_{2})$, which is shown as,
  (11) $$\begin{array}{c} E=\left(\begin{array}{c} Y_{1}\\ Y_{2} \end{array}\right), \end{array}$$
  where $Y_{1}$ and $Y_{2}$ denotes the input images of types i=1 and i=2; *E* is the output image with dimension (2·l,1).
- Classification layer: This is the final layer in our CNN model. It is a fully-connected layer with one neuron per each of defect type (i.e., E). We have six defect types in this work, the probability of having each defect type is computed as the log-likelihoods of each class. We represent it as CL(E(k,1),j). First, we apply the FC layer step to reduce the dimension to a vector of *j* neurons (j=6, the number of defect types). *Q* is a vector with dimension (j,1) which is used to reduce the dimensions of matrix *E* given in Equation (12).
  (12) $$\begin{array}{c} Q=AE+d, \end{array}$$
  where *E*, *A*, and *d* denote the input matrix with dimension (k,1), the random matrix with dimension (j,k), and the random vector of dimension (j,1), respectively. Finally, to have the log likelihood of each class, we apply the LogSoftmax function:
  (13) $$\begin{array}{c} T=log(\frac{exp(Q_{i})}{\sum_{i}exp\left(Q_{i}\right)}),i=1,2,...,j. \end{array}$$
  where *T* is the vector of *j* neurons with the likelihoods values of each class. This is the final output of the CNN model.

## 5. Experiments

In this section, we first explain the dataset used in the evaluation and the evaluation setup. Following this, the comparison baselines and metrics are introduced. Hereafter, a comparative summary of the performance evaluation is presented.

### 5.1. Dataset

**Figure 3:**
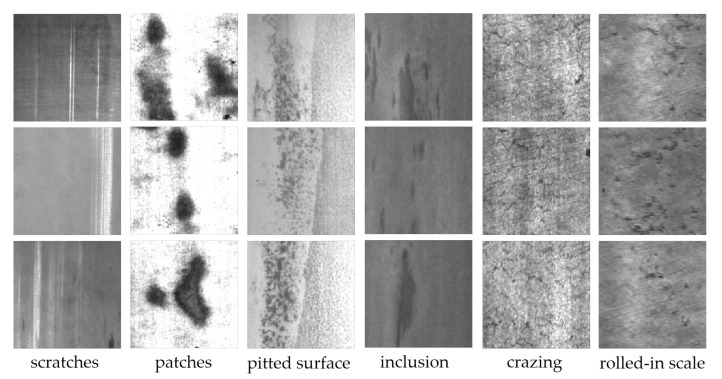
Samples of six types of the defects on the surfaces from the database.

The NEU surface defect (NEU-CLS) dataset published by Song et al. [25] was mainly used in our experiments to evaluate the performance of ConCNN and some state-of-art models. NEU-CLS dataset contains six types of defects in total, i.e., scratch(Sc), patch (Pa), pitted surface (Ps), inclusion (In), crazing (Cr), and rolled-in scale (Rs). Each defect type has 300 images with a resolution of 200×200 pixels. A total of 1800 grayscale images are present. Figure 3 shows the samples of six kinds of typical surface defects.

To verify the ConCNN’s performance on a small training dataset, we randomly select 20% of the NEU-CLS dataset as the training dataset and the rest of the samples are used as the testing dataset. In our work, a small training dataset (60 images per defect type) are employed to train models. The performance of the models based on larger training dataset, i.e., 50% as well as 80% are also compared. All results are obtained by averaging 20 runs. An additional public dataset (DAGM 2007 [26] including 6 defect types) is also used to verify the performance of ConCNN with 20% training data.

### 5.2. Setup

The AdamW optimiser [27] and the cross-entropy loss function are selected for the ConCNN with six layers (see Section 4.2). We run all simulations on a server, with Ubuntu 18.04.5 LTS operation system, Intel Core i7-8700K CPU, 63 GB RAM, and one GeForce GTX 1080 Ti GPU. We utilize Python 3.6 and some python libraries, for example, Pytorch and Scikit Learn, for all experiments. We implement all models in the same environment. The models’ parameters are set as: the initial learning rate is 0.001, learning rate decay multiplier is 0.1, the batch size is 32, and the number of epochs is 100.

### 5.3. Metrics

Two metrics are employed to measure the models’ performance, i.e., accuracy and latency.

- Accuracy: It is the ratio of the number of samples, whose prediction results are the same as the ground truth, to the total number of samples. It reflects the correctness of the classification systems.
- Latency: It is the time cost from inputting an image into the model to obtaining the prediction result.

### 5.4. Baselines

In this part, some state-of-the-art models for surface defect classification are chosen to evaluate ConCNN’s performance.

- MobileNet [28]: It is a light weight deep learning model with a streamlined architecture based on depth-wise separable convolutions.
- ThumbNet [29]: ThumbNet is an unified framework that can drastically reduce computation and memory space comparing with normal deep learning models. Since input images of a CNN have much redundancy, ThumbNet can accelerate and compress CNN models by infer thumbnail images.
- VGG 16 [30]: VGG 16 is deep learning model with 12 convolutional layers and 3 FC layers. It has a deep architecture with very small (3 × 3) convolution filters. The final layer is the soft-max layer. All hidden layers are equipped with the ReLU function to increase non-linearity.
- PyramidNet [3]: A network architecture that applied different levels of Gaussian blur to images prior to processing them with three separate VGG 16 networks. Similar to the VGG 16 approach.
- ConCNN-single: It is a single CNN model with the same architecture and parameters as the ConCNN to classify surface defects.

## 6. Results and Analysis

In this part, the performance of ConCNN is presented firstly with 60 images (20% training data) from each class for training. Table 1 illustrates the confusion matrix, where Sc, Pa, Ps, In, Cr, and Rs represent scratch, patch, pitted surface, inclusion, crazing, and rolled-in scale. We observe that the classification performance for Cr is the best (100%) while Ps has the lowest accuracy for defect classification (∼95.83%). The classification abilities of ConCNN for In, Rs, Sc, and Pa are close. The overall defect classification accuracy is about 99%.

Then, the comparison results of ConCNN with state-of-the-art are presented in detail with 60, 150, and 240 images (20%, 50%, and 80% training data) in Table 2 where Acc is short for Accuracy and tr is short for training. From this table, we can see that with the least training data ConCNN achieves the highest accuracy (about 98.89%) among all models. This is because that ConCNN benefits from the multiple-image scales information and the design of CNN fusion strategy. The accuracy of MobileNet is the worst (80.44%), the performance of ThumbNet (90.29%) and CNN (92.14%) is also not satisfying (8.70% and 5.64% lower than ConCNN) although ThumbNet has the best performance of latency (0.57 and 1.12 ms for GPU and CPU respectively). With more training data the accuracy of MobileNet, ThumbNet, and ConCNN-single increase obviously, which means that these models are dependent on the amount of training data.

The accuracy of VGG 16 (98.44%) and PyramidNet (98.68%) are close but the latency for both GPU and CPU is higher. The latency of VGG 16 with GPU and CPU is about 2.21 ms and 18.04 ms higher than that of ConCNN. Besides, PyramidNet’s latency is about 30.35 ms with GPU and about 376.13 ms with CPU, which is four times and three times higher than ConCNN, respectively. Thus, both of them are not suitable for latency-sensitive applications, such as the real-time surface defect classification, due to long latency. With more training data the accuracy increases, but due to longer latency, the training time for both models is longer. With the 20% labeled data as training dataset, the performance of [7] accomplishes 99.81% accuracy, which is slightly higher than ConCNN. However, a total 70% data including a large amount of unlabeled data is required for constructing the training data by [7] to achieve a good performance, which results in a much higher training cost. ConCNN can achieve a good trade-off between cost and performance.

It is noticeable to mention that all the presented models can predict steel strips’ defects to some extend. MobileNet uses a special convolutional operation (i.e., deep separable convolutional) which reduces the latency while does not improves accuracy. ThumbNet reduces the latency significantly by using images containing less information, which leads to low accuracy. PyramidNet achieves the highest accuracy among the state-of-the-art models since it leverages three VGG sub-networks in sequence to improve the accuracy performance, resulting in high latency. Besides, the performance of three scales with 20% training data is 99.47% ± 0.29%, which is slightly better than that of two scales but with high computation cost.

The performance of ConCNN on the DAGM dataset [26] is tested with only 20% of the training dataset, and the accuracy is 99.89% ± 0.20%. The performance is better than that in [7] when 70% labeled data is used in their model, and also better than the other related models [31].

## 7. Conclusions

The quality of steel strips is important since they are widely used in various industries, ranging from heavy industry and shipbuilding to construction and metal forging. In this paper, a light-weighted defect inspection method, named ConCNN, is proposed. ConCNN leverages the advantages of various image scales and a fusion strategy, for example, the images with a large scale improve the performance of small defect classification. ConCNN can be employed in both offline and online stages. In the online stage, ConCNN learns new incoming defect types in parallel, which further help to detect more defect types. Besides, it detects multiple types of defects accurately in real-time using a small number of training samples, which lessens the storage and labeling cost significantly. Simulation results demonstrate that ConCNN achieves 98.89% accuracy for defect classification with 5.58 ms latency and low training cost. In the future, we will improve our ConCNN by adding the function of localizing the positions of the detected defects.

## Figures and Tables

**Table 1:** Confusion metrics of Concurrent Convolutional Neural Network (ConCNN) (20% training data).

	Sc	Pa	Ps	In	Cr	Rs
Sc	239	0	2	1	0	0
Pa	0	238	0	0	0	0
Ps	0	0	230	1	0	0
In	0	0	4	238	0	0
Cr	1	0	0	0	240	1
Rs	0	2	4	0	0	239

**Table 2:** Comparison results over various number images in training dataset.

Model	Acc (20% tr/%)	Acc (50% tr/%)	Acc (80% tr/%)	Latency (GPU/ms)	Latency (CPU/ms)
MobileNet [28]	80.44 ± 0.98	83.17 ± 0.75	96.05 ± 0.79	4.68 ± 0.17	75.62 ± 1.55
ThumbNet [29]	90.29 ± 0.93	94.53 ± 0.91	96.78 ± 0.62	**0.57 ± 0.09**	**1.12 ± 0.32**
VGG 16 [30]	98.44 ± 0.43	**99.56** ±**0.23**	**99.67± 0.32**	7.79 ± 0.35	108.62 ± 23.17
PyramidNet [3]	98.68 ± 0.18	99.42 ±0.20	99.56 ±0.28	30.35 ± 0.66	376.13 ± 38.42
ConCNN-single	92.14 ± 0.98	94.68 ± 0.95	95.36 ± 0.83	3.08 ± 0.17	47.79 ± 1.55
ConCNN	**98.89 ± 0.42**	99.47 ± 0.15	99.63 ± 0.54	5.58 ± 0.26	90.58 ± 4.87

## Abbreviations

The following abbreviations are used in this manuscript:

CNN	Convolutional Neural Network
ConCNN	Concurrent Convolutional Neural Network
ConCNN-single	ConCNN with only a single CNN model
ReLU	Rectified Linear Units
DCNNs	Deep Convolutional Neural Network
FC	Fully Connected
CL	Classification Layer
NEU-CLS	NEU Surface Defect
Sc	scratch
Pa	patch
Ps	pitted surface
In	inclusion
Cr	crazing
Rs	rolled-in scale
Acc	Accuracy
tr	training
